# Blockade of the Programmed Death-1 (PD1) Pathway Undermines Potent Genetic Protection from Type 1 Diabetes

**DOI:** 10.1371/journal.pone.0089561

**Published:** 2014-02-28

**Authors:** Nora M. Kochupurakkal, Annie J. Kruger, Sudipta Tripathi, Bing Zhu, La Tonya Adams, Daniel B. Rainbow, Aldo Rossini, Dale L. Greiner, Mohamed H. Sayegh, Linda S. Wicker, Indira Guleria

**Affiliations:** 1 Transplantation Research Center, Brigham and Women's Hospital and Children's Hospital Boston, Harvard Medical School Renal Division, Boston, Massachusetts, United States of America; 2 Program in Molecular Medicine, University of Massachusetts Medical School, Worcester, Massachusetts, United States of America; 3 Center for Neurologic Diseases, Brigham and Women's Hospital, Boston, Massachusetts, United States of America; 4 Cambridge Institute for Medical Research, University of Cambridge, Cambridge, United Kingdom; Dasman Diabetes Institute, Kuwait

## Abstract

**Aims/Hypothesis:**

Inhibition of PD1-PDL1 signaling in NOD mice accelerates onset of type 1 diabetes implicating this pathway in suppressing the emergence of pancreatic beta cell reactive T-cells. However, the molecular mechanism by which PD1 signaling protects from type 1 diabetes is not clear. We hypothesized that differential susceptibility of *Idd* mouse strains to type 1 diabetes when challenged with anti PDL1 will identify genomic loci that collaborate with PD1 signaling in suppressing type 1 diabetes.

**Methods:**

Anti PDL1 was administered to NOD and various *Idd* mouse strains at 10 weeks of age and onset of disease was monitored by measuring blood glucose levels. Additionally, histological evaluation of the pancreas was performed to determine degree of insulitis. Statistical analysis of the data was performed using Log-Rank and Student's t-test.

**Results:**

Blockade of PDL1 rapidly precipitated type 1 diabetes in nearly all NOD *Idd* congenic strains tested, despite the fact that all are moderately (*Idd5, Idd3* and *Idd10/18*) or highly (*Idd3/10/18* and *Idd9*) protected from spontaneous type 1 diabetes by virtue of their protective *Idd* genes. Only the *Idd3/5* strain, which is nearly 100% protected from spontaneous disease, remained normoglycemic following PDL1 blockade.

**Conclusions:**

These results indicate that multiple *Idd* loci collaborate with PD1 signaling. Anti PDL1 treatment undermines a large portion of the genetic protection mediated by *Idd* genes in the NOD model of type 1 diabetes. Basal insulitis correlated with higher susceptibility to type 1 diabetes. These findings have important implications since the PD1 pathway is a target for immunotherapy.

## Introduction

Type 1 diabetes is a multi-factorial autoimmune disease resulting from the destruction of pancreatic beta cells by autoreactive T cells. Both environmental factors and variations in multiple genetic loci have been implicated in the etiology of type 1 diabetes. The NOD mouse recapitulates many features of human type 1 diabetes and is used extensively as an experimental model.

Programmed death-1 (PD1) and its ligand PDL1 have been shown to play an important role in regulating T cell activation and peripheral tolerance. The PD1- PDL1 pathway is being explored for developing therapies against recurrent solid tumors and infectious diseases (such as HIV), since blocking the pathway results in an increased immune response against tumors and infections [1]–[3].

We and others have shown that PD1-PDL1 interaction is critical for the regulation of CD4 and CD8 autoreactive T cells involved in the development of type 1 diabetes [4], [5]. Further, while PD1 deficiency resulted in lupus-like symptoms in C57BL6 or BALB/c mice, it led to accelerated onset and frequency of type 1 diabetes in NOD mice [6].

In the NOD mouse model, blockade of PD1-PDL1 pathway results in accelerated onset of autoimmune diabetes, raising concern that immunotherapy by such blockade could increase susceptibility to autoimmune diseases, particularly in individuals harboring susceptibility alleles. To date, numerous MHC-linked and non-MHC-linked genes and genetic regions influencing the susceptibility to autoimmune diseases have been identified in humans, rats and mice. In insulin dependent type 1 diabetes, many genes implicated in the control of glycemia have also been described in the NOD *Idd* congenic mouse strains. Congenic NOD strains have genetic loci from diabetes resistant parental strains inserted (introgressed) into their genome (reviewed in [7]).

In recent years, NOD H2-Ag7 and H2-Enull MHC class II genes have been unequivocally identified as susceptibility genes within *Idd1* [8]. Additionally, accumulated data support the existence of particular susceptibility genes within other *Idd* regions.

*Idd3* is the most well studied *Idd* region [9]–[11]. Protective alleles in *Idd3* reduce type 1 diabetes frequency and *Il2* and *Il21* are the prime candidate genes. The protective effects of *Idd3* are evident in multiple cell types including antigen-presenting cells, effector T cells and regulatory (FoxP3^+^) T cells which are critical for maintaining immune cell homeostasis [12], [13].

The prime gene candidate for *Idd10* is *Cd101* whose expression on regulatory T cells and dendritic cells is affected in NOD/B6 polymorphisms [14]. *Vav3*, which encodes a guanine nucleotide exchange factor important for signaling in immune cells, is the only complete gene present in the 604 kb *Idd18.1* region on Chromosome 3. Gene expression evidence indicates that alteration of *Vav3* expression is an etiological factor in the development of autoimmune beta-cell destruction in NOD mice, making it the most likely candidate [15]. The *Idd5* region is composed of at least 5 sub-regions. *Idd5* contributes to islet-specific CD8 T cell tolerance and to loss of CD4 tolerance through both lymphocytic and non-lymphocytic compartments [9], [16], [17]. Candidate genes for *Idd5* sub regions include *Ctla4* for *Idd5.1* [18], *Slc11a1* for *Idd5.2* [19] and *Acadl* for *Idd5.3* [20]. The *Idd9* region on chromosome 4 is composed of at least three separate intervals, *Idd9.1*, *Idd9.2*, and *Idd9.3* and numerous candidate genes are present. Fine mapping of type 1 diabetes regions *Idd9.1* and *Idd9.2* revealed further genetic complexity [21]. The *Idd9.1* sub-region has been shown to influence regulatory T cells and iNKT cells [22], [23]. *Idd9.2* and *Idd9.3* have been linked to limit the expansion of islet specific autoreactive CD8 T cells [24]. The *Idd9.3* candidate gene encodes 4-1bb, which is important for CD4 and CD8 T cell activation [25]. The *Idd9* locus has also been previously described to play a role in homing of islet-specific T cells [26]. Overall, *Idd9* mice display profound resistance to diabetes even though nearly all develop insulitis.

In this study, we made use of four loci on Chromosome 3, four on Chromosome 1, and three on Chromosome 4 to determine which *Idd* regions conferring resistance to type 1 diabetes remain so in the presence of anti PDL1 negative co-stimulatory blockade. We show that blockade of the PD1-PDL1 interaction results in accelerated onset of type 1 diabetes in all the NOD *Idd* strains except NOD *Idd3/5*. Additionally, basal insulitis levels correlated with higher susceptibility to type 1 diabetes induction by anti PDL1 treatment.

## Methods

### Mice

Female NOD mice were obtained from Taconic (Germantown, NY, USA). NOD congenics were obtained through the Taconic Emerging Models program; NOD.B10-*Idd9.1/9.2/9.3* (line 905) [27], NOD.B10-*Idd9.1* (line 1565) [22], NOD.B10-*Idd9.2* (line 1566) [22], NOD.B10-*Idd9.3* (line 1106) [22], NOD.B6-*Idd10/18* (line 7754) [14], [15], [27]–[30], NOD.B10-*Idd5.1 Idd5.2 Idd5.3* (line 1094) [31], NOD.B10-*Idd5.1* (line 2193) [31], NOD.B10-*Idd5.2* (line 6146) [31], NOD.B10-*Idd5.3* (line 6360) [32], NOD.B6-*Idd3/10/18* (line 1538) [15], [27], NOD.B6-*Idd3* (line 1098) [12], [27], NOD.B6-*Idd3* B10-*Idd5* (line 6109) [27], NOD.B10-*Idd5.2 Idd5.3* (line 1595) [32] and NOD.B10-*Idd5.2 Idd5.3* Idd3 (lines 7380 and 9245, data combined in this study)[33]. The NOD congenic strains will be referred to by their *Idd* numbers without adding NOD before the designated *Idd* region. When referring to congenic mice containing two or more *Idd* loci, the loci will be separated by slashes. For example, *Idd9.1 Idd9.2 Idd9.3* (line 905) mice will be referred to as *Idd9.1/9.2/9.3* for simplicity. Spontaneous development of diabetes in females from these strains of mice has been published (references noted above). BDC2.5 TCR Tg mice were a gift of Drs. Diane Mathis and Christophe Benoist [34]. NY8.3 mice were obtained from JDRF's Resource Sharing Program. All mouse experiments were approved by the Institutional Animal Care and Use Committee of Children's Hospital Boston and University of Massachusetts Medical School. All mice were cared for in accordance with Boston Children's Hospital and the University of Massachusetts Medical School institutional guidelines.

### Antibodies and Treatment Protocol

Anti mouse PDL1 mAb (MIH6, rat IgG2a) was generated as previously described, [35] and was manufactured by BioXCell (West Lebanon, NH, USA). Rat IgG (Sigma-Aldrich, St.Louis, MO, USA) served as a control. Anti PDL1 was injected in PBS i.p.; 500 µg on day 0, followed by 250 µg on days 2, 4, 6, 8, and 10 unless indicated otherwise. Mice were 10 weeks of age at the start of treatment.

### Monitoring for Diabetes

The onset of type 1 diabetes was defined as a random blood glucose reading of 250 mg/dl or greater for three consecutive days. Blood glucose levels were measured daily for the first two weeks followed by 2–3 times per week by One Touch Ultra meter and One Touch Ultra test strips (LifeScan, Milipitas, CA, USA).

### Histology

Pancreases were fixed in 10% neutral buffered formalin in PBS for 16 h and transferred to 70% ethanol before being embedded in paraffin. Tissue sections were stained with H&E (Dana Farber Cancer Institute's Research Pathology Core, Boston, MA, USA) and insulitis was graded by scoring a minimum of 10 islets per mouse. Each mouse received a score from the average of graded islets. Scores were defined as: 0 -no insulitis, 1 –peri-insulitis, 2<50% insulitis, 3>50% insulitis, 4 -100% insulitis.

### Adoptive transfer of BDC2.5 TCR-transgenic cells

Anti-CD25 mAb (clone 7D4, ATCC, Manassas, VA, USA) and rabbit complement (Cedarlane, Burlington, NC, USA) were incubated with splenocytes of BDC2.5 TCR Tg mice at 37°C for 45 min to remove CD25^+^ cells (technique described in [36]. Remaining cells were labeled with 7.5 µM CFSE (Invitrogen, Carlsbad, CA, USA) according to the manufacturer's instructions. The percentage of CD4^+^ T cells in the splenocyte suspension was determined by flow cytometry to calculate the volume needed for injection of 0.5×10^6^ BDC2.5tg CD4^+^ T cells. Splenocytes were labeled with CD3, CD4 and Vβ4 antibodies and analyzed by flow cytometry. Half a million CD4^+^ T cells were injected i.v. into the tail veins of pre-diabetic 8–10 week old female NOD and age matched *Idd3/10/18* mice. Mice received 500 µg of either anti PDL1 mAb or rat IgG one day before transfer (day 0), and 250 µg on days 2 and 4. Pancreatic LN and spleens were harvested on day 6 and cells were stained for CD4, Vβ4 (KT4, BD Biosciences, USA), labeled with CFSE and analyzed by flow cytometry.

### Adoptive transfer of NY8.3 TCR transgenic cells

Splenocytes from NY8.3-NOD TCR Tg mice were used for adoptive transfer studies. Splenocytes were enriched for CD8^+^ T cells using the CD8^+^ T cell untouched isolation kit II (Miltenyi, Auburn, CA, USA). One million CD8^+^ T cells were injected i.v. into the tail veins of pre-diabetic 8–10 week old female NOD and age matched *Idd3/10/18* mice. The recipients received 500 µg of either anti PDL1 mAb or IgG Ab one day before transfer (day 0), and 250 µg on days 2 and 4. The pancreatic lymph node and spleen were harvested on day 6 and the cells were acquired by flow cytometry for CFSE labeling.

### RNA extraction and Real time PCR of pancreas tissue

Pancreas tissue from *Idd9* mice was stored in RNAlater solution (Ambion, Austin, TX, USA) and total RNA was extracted using the RNAeasy Mini kit (Qiagen, Gaithersburg, MD, USA). RNA was redissolved in RNAse-free water and the yield quantified by spectrophotometry. Equal amounts of RNA were used for quantitative real time PCR. First strand cDNA synthesis was performed using Superscript III (Invitrogen, Grand Island, NY, USA). All reactions were run in triplicates in an ABI Prism 7300 (Applied Biosystems, Foster City, CA, USA) and normalized to GAPDH. For a list of primers used, see Electronic Supplemental Material.

### Statistical Analysis

Kaplan-Meier survival analysis was performed to compare the frequency of diabetes in sub-congenic strains using the Log-Rank test. Differences in insulitis between congenic strains were analyzed by unpaired two-tailed Student's t test. A p value of <0.05 was considered significant.

## Results

### Diabetes-resistant NOD Idd strains develop diabetes upon anti PDL1 treatment

In order to determine if blocking the PD1-PDL1 pathway would induce autoimmune diabetes in mice genetically protected from developing the disease, strains of mice protected from type 1 diabetes because they carry protective genes derived from B6 and B10 mice, were treated with anti PDL1 mAb. We tested the following 14 NOD congenic strains to examine the genetic protection due to a range of genes and gene combinations which can possibly contribute to resisting the precipitation of type 1 diabetes following PDL1 blockade: *Idd3*, *Idd10/18*, *Idd3/10/18*, *Idd5* (which includes the subcongenic regions of *Idd5.1*, *Idd5.2*, and *Idd5.3*), *Idd5.1, Idd5.2, Idd5.3*, *Idd5.2/5.3*, *Idd3/5, Idd3/5.2/5.3, Idd9* (which includes the sub-congenic regions of *Idd9.1*, *Idd9.2* and *Idd9.3*), *Idd9.1*, *Idd9.2* and *Idd9.3*. The incidence of diabetes for females from these 14 strains at 28 to 30 weeks of age are <5% (*Idd3/10/18, Idd3/5, Idd3/Idd5.2/Idd5.3* and *Idd9*), 15–40% (*Idd3, Idd9.1* and *Idd5*), and 45–65% (*Idd5.1, Idd5.2, Idd5.3, Idd5.2/Idd5/3*, *Idd9.1, Idd9.2, Idd9.3*, and *Idd10/18*). Throughout the time period of defining the *Idd* regions using congenic strains that are resistant to type 1 diabetes (1990 to 2010) the NOD female diabetes incidence has ranged from 70–90% at 28 to 30 weeks of age.

### Idd5

**Figure 1:**
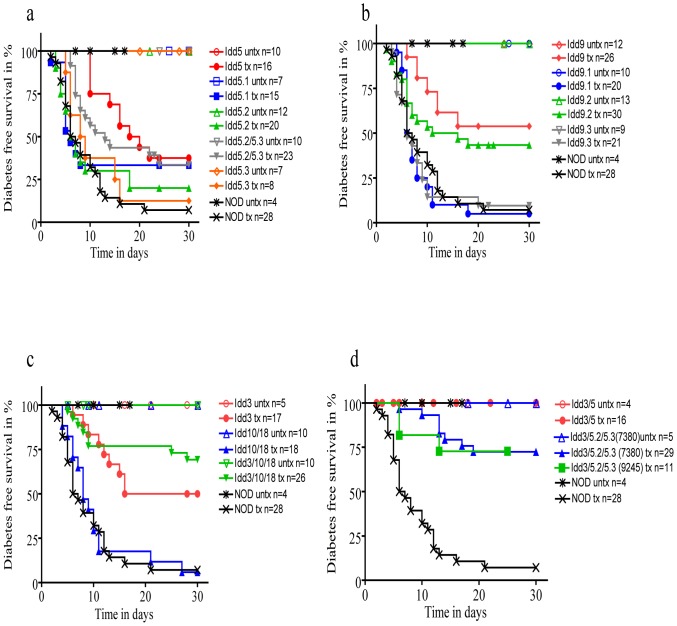
Incidence of diabetes in NOD congenic mouse strains undergoing anti PDL1 treatment. Treatment was started at 10 weeks of age. a): Incidence of diabetes in *Idd5* and sub-congenics *Idd5.1*, *Idd5.2*, *Idd5.3* and *Idd5.2*/*5.3* until day 30 after anti PDL1 treatment. In *Idd5* mice (n = 16) 62.5% developed diabetes, in *Idd5.1* (n = 15) 66.6%, in *Idd5.2* (n = 20) 80%, in *Idd5.3* (n = 8) 87.5% and in *Idd5.2*/5.3 (n = 23) 66.5%. NOD mice (n = 28) had a 92.5% incidence of diabetes by day 30. All control treated mice did not develop diabetes. b): In *Idd9* (n = 26) 46.15% developed diabetes, in *Idd9.1* (n = 20) 95%, in *Idd9.2* (n = 30) 56.6% and in *Idd9.3* (n = 21) 90.5%. c): *Idd3* (n = 17) developed diabetes at a rate of 50%, *Idd10/18* (n = 18) at 94.1%, *Idd3/10/18* (n = 26) at 30.8%. d): In *Idd3/5* (n = 16) 0% of anti - PDL1 treated mice developed diabetes, in *Idd3/5.2*/5.3 (n = 13) 15.4%. Statistics and cumulative incidence for the strains are shown in separate Tables for Figure 1. P-values were calculated using Log-rank (Mantel-Cox test).

Untreated *Idd5* mice have a 40% cumulative incidence of diabetes at 28 to 30 weeks of age [37]. With anti PDL1 treatment, 10-week old *Idd5* mice started to develop the disease by day 10, and after 30 days, 62.5% had developed type 1 diabetes (Figure 1a, Table 1, 2). The sub-congenic strains *Idd5.1, Idd5.2*, *Idd5.2*/*5.3* and *Idd5.3* showed a faster onset of disease from day 3 to day 7. The *Idd5.3* strain had the highest cumulative incidence of type 1 diabetes following anti PDL1 treatment, with 90% of the mice developing disease by day 30 (P = 0.0140 *Idd5* vs *Idd5.3*), followed by *Idd5.2* with 80% cumulative incidence (P = 0.0194 *Idd5* vs *Idd5.2*). *Idd5.1* developed diabetes with a 66% cumulative incidence. The combination of two sub-congenic strains in *Idd5.2*/*5.3* developed type 1 diabetes with a cumulative incidence of 65% (Figure 1a, Table 1, 2). Of the control NOD mice treated with anti PDL1 93% developed type 1 diabetes by day 21. None of the control NOD mice developed type 1 diabetes during the course of the experiment (Figure 1a, Table 1, 2).

**Table 1:** Statistical Significance Figure 1.

Comparison of *Idd* Strains	p-value
*Idd5* vs. *Idd5.1*	p = 0.1679
*Idd5* vs. *Idd5.2*	p = 0.0194
*Idd5* vs. *Idd5.3*	p = 0.0140
*Idd5.1* vs. *Idd5.2*/5.3	p = 0.0539
*Idd9* vs. *Idd9.1*	p<0.0001
*Idd9* vs. *Idd9.2*	p = 0.2029
*Idd9* vs. *Idd9.3*	p<0.0001
*Idd9.2* vs. *Idd9.1*	p = 0.094
*Idd9.2* vs. *Idd9.3*	p = 0.0118
*Idd3* vs. *Idd10/18*	p = 0.0103
*Idd3/10/18* vs.	p<0.0001
*Idd10/18*	
*Idd3* vs. *Idd3/10/18*	p = 0.2531
*Idd3/5* vs *Idd3/5.2*/5.3	p = 0.2298

**Table 2:** Spontaneous incidence of type 1 diabetes at 7 months of age compared to anti PDL1 treatment of 10 week old mice.

Strain	Line	Cumulative Incidence of spontaneous type 1 diabetes	Cumulative Incidence of type 1 diabetes with aPDL1 treatment starting at 10 weeks
NOD.B10-*Idd5.1*	1094	40%	62.5%
*Idd5.2 Idd5.3*			
NOD.B10-*Idd5.1*	2193	62%	66%
NOD.B10-*Idd5.2*	6146	38%	80%
NOD.B10-*Idd5.3*	6360	69%	90%
NOD.B10-*Idd5.2*	1595	25%	65%
*Idd5.3*			
NOD.B10-	905	3%	46%
*Idd9.1/9.2/9.3*			
NOD.B10-*Idd9.1*	1565	35%	95%
NOD.B10-*Idd9.2*	1566	55%	56%
NOD.B10-*Idd9.3*	1106	50%	90%
NOD.B6-*Idd3*	1098	20%	50%
NOD.B6-	7754	50%	94%
*Idd10/Idd18*			
NOD.B6-*Idd3/10/18*	1538	9%	31%
NOD.B6-*Idd3* B10-	6109	1%	0%
*Idd5*			
NOD.B6-*Idd3*	7380	0%	15%
*Idd5.2/5.3*			
NOD.B6-*Idd3*	9245	0%	
*Idd5.2/5.3*			
NOD		70–90%	93%

### Idd9, Idd9.1, Idd9.2, Idd9.3

*Idd9* mice receiving anti PDL1 treatment developed type 1 diabetes with a cumulative incidence of 46% between days 6 and 16. The sub-congenic strain *Idd9.2* showed a reduced cumulative incidence of diabetes at 56% (between days 4–18), whereas the *Idd9.1* and *Idd9.3* strains had a much higher cumulative incidence with 95% and 90% respectively (onset from day 3 to day 22), which is quite similar to 93% type 1 diabetes in anti PDL1 treated NOD mice (between days 4–12) (Figure 1b, Table 1, 2). As the *Idd9.2* strain had the lowest cumulative incidence among *Idd9* subcongenic strains, we deduced that this sub-congenic strain must be associated with the protective allele in the *Idd9* congenic interval.

### Idd3, Idd10/18 and Idd3/10/18

Twenty percent of *Idd3* mice spontaneously develop type 1 diabetes within 7–8 months [37]. Upon anti PDL1 administration, 50% of the mice developed type 1 diabetes between days 6 and 16 (Figure 1c). *Idd10/18* mice have a 50% occurrence of spontaneous diabetes, and with anti PDL1 treatment 94% of mice developed the disease between days 4 to 27. The *Idd3/10/18* strain develops diabetes with 31% incidence upon anti PDL1 treatment (days 4 to 28), which is ∼6-fold greater than the spontaneous incidence at the age of 7–8 months (Figure 1c, Table 1, 2).

### Idd3/Idd5 and Idd3/5.2/5.3

The *Idd3/5* strain has protective alleles at both *Idd3* and *Idd5* and only 1% of mice develop spontaneous diabetes by 7–8 months of age [38]. Anti PDL1 treatment did not induce diabetes in *Idd3/5* mice as 100% of them stayed non-diabetic over the course of 30 days PDL1 blockade (Figure 1d). The *Idd3/5.2*/5.3 (without protective alleles at the *Idd5.1* sub-region) strain that is also almost completely protected from spontaneous diabetes shows susceptibility to treatment with anti PDL1, and 15% of the mice developed diabetes by day 30 (Figure 1d, Table 1, 2).

### Insulitis in anti PDL1 treated congenic strains

One of the hallmarks of developing type 1 diabetes is the presence of infiltrating lymphocytes in the pancreas. *Idd* congenic strains have made it possible to identify checkpoints of disease progression. Ninety percent of *Idd9* mice show evidence of islet insulitis, but only 3% develop diabetes spontaneously [21], [37]. Analysis of insulitis scores of the *Idd* strains revealed that NOD congenics that were partially (*Idd 9.2*, *Idd 3/10/18*) or fully (*Idd3/5*) protected from anti PDL1 accelerated diabetes had lower basal insulitis scores (*Idd 9.2* (0.18±0.11), *Idd3/10/18* (0.24±0.059) and *Idd3/5* (0.0±0.0) compared to the almost unprotected *Idd9.1* (1.2±0.3), *Idd9.3* (0.82±0.14) and the NOD (1.23±0.24) mice. These results were statistically significant (***Idd9.2*** vs *Idd9.1*, *Idd9.3*, NOD p value 0.0016, 0.0031 and 0.0007, respectively; ***Idd3/10/18*** vs *Idd9.1*, *Idd9.3*, NOD p value <0.0001, 0.0002 and <0.0001, respectively; ***Idd3/5*** vs *Idd9.1*, *Idd9.3*, NOD p value 0.0047, 0.0014 and 0.0018 respectively).

**Figure 2:**
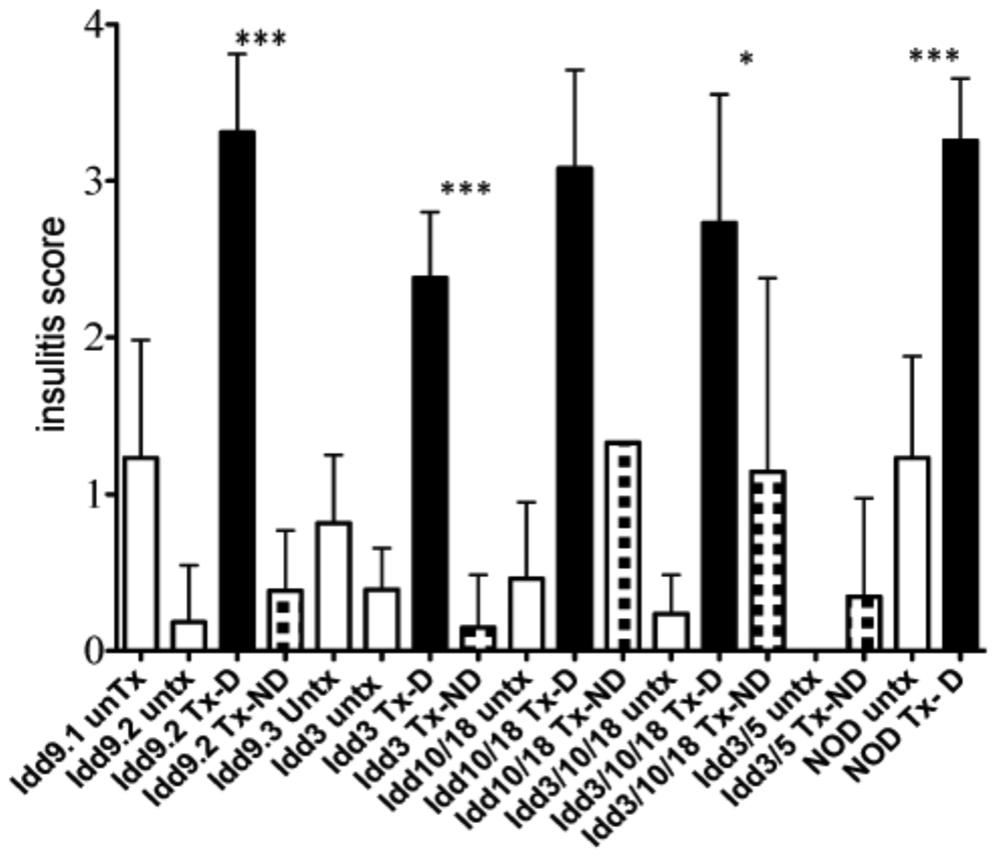
Insulitis scores in anti PDL1 treated mice. *Idd* congenic mice were grouped into control treated (untx, white columns, *Idd9.1* n = 7, *Idd9.2* n = 10, *Idd9.3* n = 9, *Idd3* n = 4, *Idd10/18* n = 5, *Idd3/10/18* n = 18, *Idd3/5* n = 3, NOD n = 7), anti PDL1 treated diabetic (Tx-D, checked columns, *Idd9.2* n = 4, *Idd3* n = 5, *Idd10/18* n = 8, *Idd3/10/18* n = 6, NOD n = 11) and anti PDL1 treated non-diabetic (Tx-ND, black columns, *Idd9.2* n = 5, *Idd3* n = 5, *Idd10/18* n = 1#, *Idd3/10/18* n = 6, *Idd3/5* n = 17) mice. The antibody treatment was given to 10-week old mice at day 0 (500 µg), and days 2, 4, 6, 8 and 10 (250 µg). The injections were stopped once the mouse had become diabetic and had a glucose reading of >250 mg/dl on two consecutive days. H&E sections of pancreases were scored for degree of infiltrating lymphocytes in islets. Results are expressed as Mean±SD. P-values are expressed as * (P<0.05), ** (P<0.01), *** (P<0.0001). # In case of the *Idd10/18* mice, treatment with anti PDL1 resulted in 94% of diabetes incidence. Therefore, it was extremely difficult to increase the n of anti PDL1 treated non-diabetic group.

Clearly, there seems to be a direct link between basal insulitis levels and the incidence of anti PDL1 induced accelerated diabetes. Interestingly, *Idd9.2* mice that turned diabetic showed similarly high insulitis scores (3.31±0.25) upon anti PDL1 treatment as treated NOD mice (3.258±0.1195), while the *Idd9.2* mice which stayed non-diabetic had almost no pancreatic infiltrates and low insulitis scores (0.38±0.17) (Figure 2).

### Cytokine and chemokine profile in Idd9 subcongenic mice

**Figure 3:**
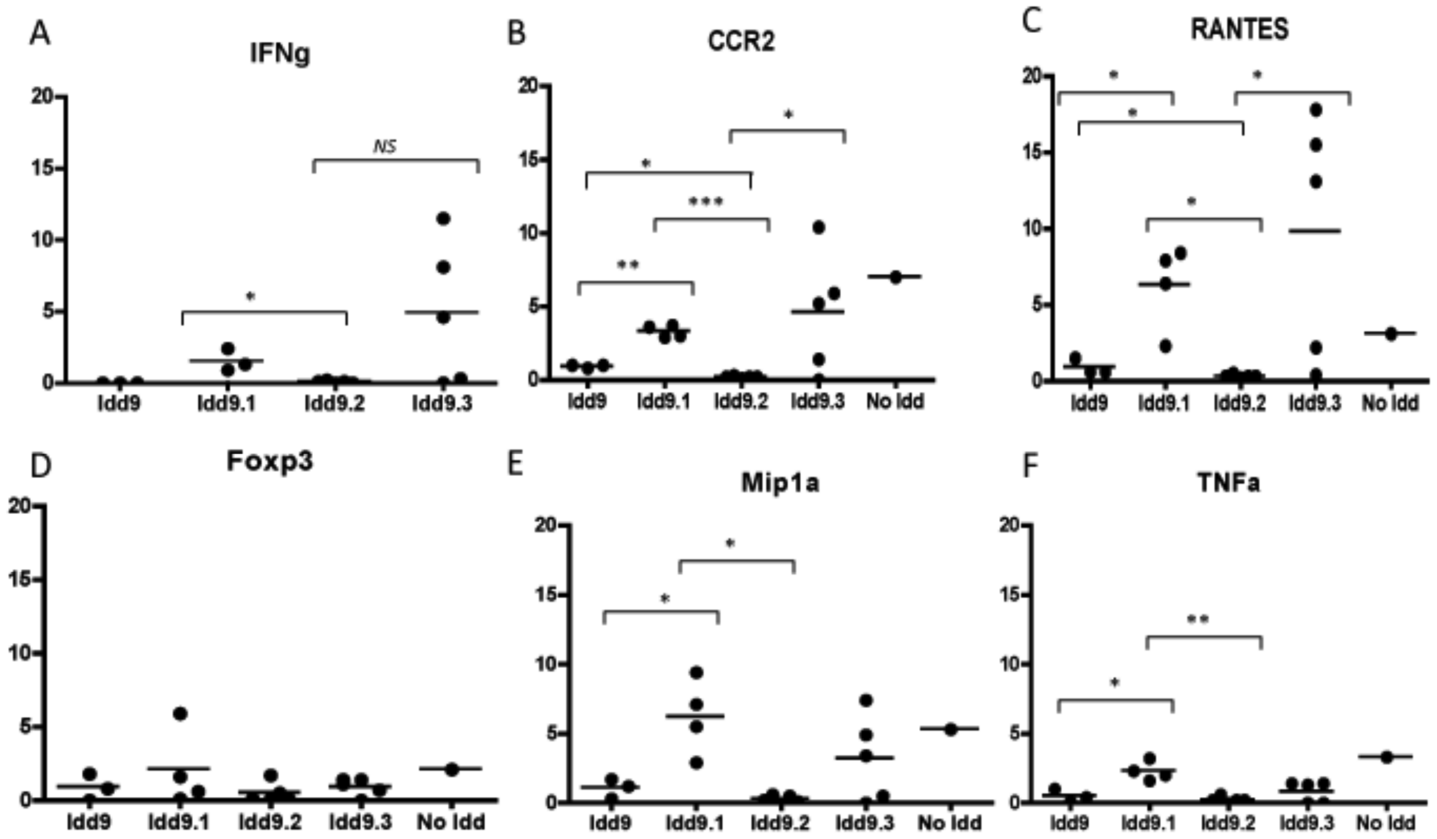
Quantitative PCR detection of cytokine, chemokine and transcription factor levels in pancreas tissue after anti PDL1 treatment in *Idd9* (n = 3) and subcongenics *Idd9.1*(n = 3), *Idd9.2* (n = 5), *Idd9.3* (n = 5). Mice (10 weeks old) were treated with 500 µg anti PDL1 on day 0 and 250 µg anti PDL1 on days 2, 4, 6, 8 and 10 by i.p. injection. Pancreas tissue was harvested on day 30 or when mice had turned diabetic. Horizontal lines show median value. P-values are expressed as * (P<0.05), ** (P<0.01), *** (P<0.0001) in figure. a): IFN-γ: *Idd9.1* vs. *Idd9.2* p = 0.0039; *Idd9.1* vs. *Idd9.3* p = 0.063. b): CCR2: *Idd9* vs. *Idd9.1* p = 0.0003; *Idd9.1* vs. *Idd9.2* p<0.0001; *Idd9.2* vs. *Idd9.3* p = 0.0431; *Idd9* vs. *Idd9.3* p = 0.0183. c): RANTES: *Idd9* vs. *Idd9.1* p = 0.022; *Idd9.1* vs. *Idd9.2* p = 0.0016; *Idd9.2* vs. *Idd9.3* p = 0.0284; *Idd9* vs. *Idd9.3* p = 0.0382. d): No significant differences in FoxP3 expression between *Idd9* and subcongenics. e): MIP-1α: *Idd9* vs. *Idd9.1* p = 0.0257; *Idd9.2* vs. *Idd9.1* p = 0.0017. f): TNF-α:*Idd9* vs. *Idd9.1* p = 0.019; *Idd9.2* vs. *Idd9.1* p = 0.0004.

The cumulative incidence of diabetes following anti PDL1 treatment for *Idd9.2* was different from that of *Idd9.1* and *Idd9.3* mice. Nearly 100% of *Idd9.1* and *Idd9.3* mice while only 56% of *Idd9.2* mice developed type 1 diabetes following anti PDL1 treatment. Basal insulitis was also lower in *Idd9.2* versus *Idd9.1* and *Idd9.3* mice. Therefore we sought to determine if any cytokines or chemokines were differentially expressed in these three sub-congenic lines. Real time PCR of pancreas tissue of anti PDL1 treated mice showed that diabetic *Idd9.2* mice had lower expression of IFN-γ, TNF-α, CCR2, RANTES (CCL5) and MIP-1α (CCL3) as compared to diabetic *Idd9.1* and *Idd9.3* mice (Figure 3a-f). MIP-1α up-regulation has been associated with progression to type 1 diabetes [39]. *Idd9* mice also had lower cytokine and chemokine levels than the *Idd9.1* and *Idd9.3* substrains. These studies show that a low level of insulitis as observed in *Idd9.2* correlates with lower levels of cytokines even when diabetes develops in some of these mice following PDL1 blockade. It remains to be seen if the quality of insulitis in *Idd9* and *Idd9.2* mice is different from that present in the *Idd9.1* and *Idd9.3* substrains.

### Proliferation of BDC2.5 Tg CD4^+^ T cells and NY8.3 CD8^+^ T cells in pancreatic LN of Idd3/10/18 mice following anti PDL1 treatment

**Figure 4:**
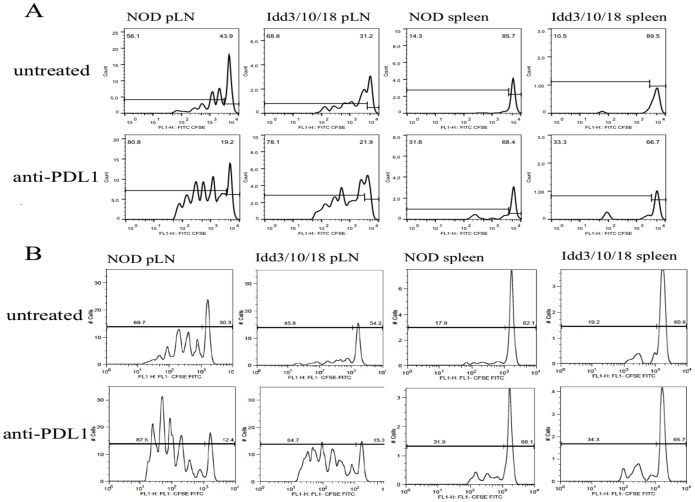
Proliferation of adoptively transferred BDC2.5 Tg CD4 T and NY8.3 tg CD8^+^ T cells in pancreatic LN and spleen of anti PDL1 treated NOD and *Idd3/10/18* mice. A) Representative CFSE dilution plot for each group is shown. Cells were gated on CD4^+^ Vbeta 4^+^. B) A representative CFSE dilution plot of transferred NY8.3tg T cells for each group is shown. Cells were gated on CD8^+^ Vbeta 8^+^.

**Figure 5:**
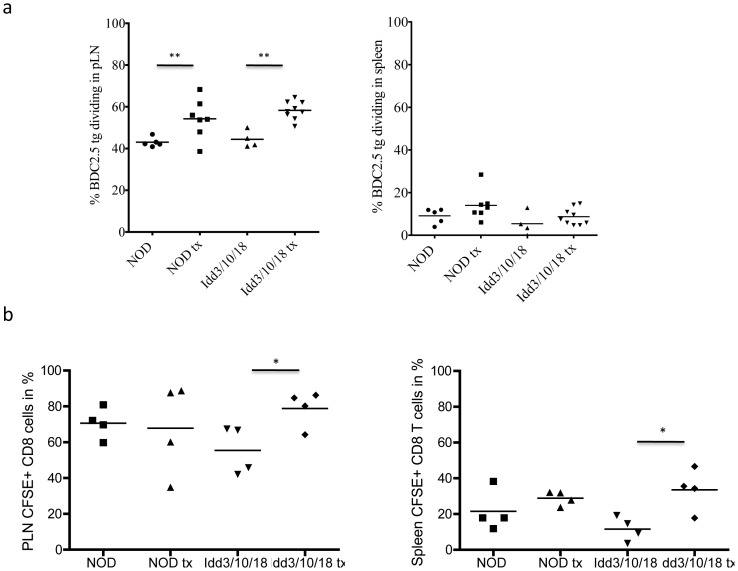
Proliferation of adoptively transferred BDC2.5 Tg CD4 T and NY8.3 tg CD8^+^ T cells in pancreatic LN and spleen of anti PDL1 treated NOD and *Idd3/10/18* mice. a) Collective data from 3 out of 8 experiments of the percentage of CFSE-dividing cells (gated on CD4^+^ Vβ4^+^) are shown. Horizontal lines express mean value. For CD4^+^ T cells pLN, NOD, control vs. treated p = 0.0276; pLN, *Idd3/10/18*, control vs. treated p = 0.0002. b) A representative experiment from 3 performed is shown. For CD8 T cells pLN, *Idd3/10/18* control vs. treated p = 0.0321, spleen *Idd3/10/18* control vs. treated p = 0.0185.

We performed an adoptive transfer of transgenic T cells and analyzed their proliferation rates to identify differences between the congenic strains undergoing anti PDL1 blockade. Adoptive transfer of CFSE-labeled BDC2.5 Tg CD4^+^ T cells into untreated NOD and *Idd3/10/18* mice showed similar proliferation rates in the pancreatic LN. With administration of anti PDL1, both NOD (P = 0.0276) and *Idd3/10/18* (P = 0.0002) strains showed significantly higher proliferation of BDC2.5 Tg T cells. No difference was detected in BDC2.5 Tg CD4^+^ proliferation between untreated and anti PDL1 treated NOD and *Idd3/10/18* mice (Figure 4a, 5a). In another set of experiments we adoptively transferred CFSE labeled CD8^+^ 8.3 TCR Tg^+^ T cells in *Idd3/10/18* mice (Figure 4b, 5b) and analyzed their rate of proliferation following anti PDL1 treatment. NY8.3 CD8^+^ Tg^+^ T cells divided more frequently as portrayed by an increase in the number of CFSE-diluted CD8^+^ T cells in *Idd3/10/18* mice that received anti PDL1 antibody as compared to mice that received control IgG. These data show that both auto-reactive CD4 as well as CD8 T cells expand in *Idd3/10/18* mice following anti PDL1 treatment, similar to that of untreated and anti PDL1 treated NOD mice.

## Discussion

More than 38 *Idd* regions from resistant strains that confer protection in the NOD model, have been described to date [7], [40]. In the current study we examined the effects of 9 *Idd* regions, alone and in combination, on accelerated type 1 diabetes following anti PDL1 treatment. These congenic and subcongenic mice have multiple protective alleles that mediate varying degrees of resistance to type 1 diabetes. We chose the NOD *Idd* congenics (*Idd3/5, Idd9, Idd3/10/18 and Idd3/5.2/5.3*) that are almost completely protected from spontaneous diabetes occurrence due to allelic interactions between the candidate genes present in the loci and subloci. We also looked at mouse strains that are variations of the above-mentioned strains, containing the individual locus or a combination of loci, where the disease progression is either moderate (*Idd3, Idd9.1, Idd5*) or relatively high but always lower than that of the NOD parental strain (*Idd5.1, Idd5.2, Idd5.3, Idd5.2/5.3, Idd9.1, Idd9.2, Idd9.3, Idd10/18*). Frequency of diabetes in these mice strains range from <5% to 65%.

PDL1 blockade is known to accelerate diabetes precipitation in NOD mice. Our aim was to determine if the interaction between the protective *Idd* loci in the different NOD congenics affects diabetes induction by PDL1 blockade. All except one among the NOD congenic strains tested here, *Idd3/5*, developed accelerated type 1 diabetes following anti PDL1 treatment. Our data show that PDL1 blockade is not enough to induce accelerated diabetes in the NOD *Idd3/5* congenic mice strain that contains alleles for *Il2, Ctla4, Slc11a1* and *Acadl*. The interaction between these alleles is able to protect the mice from diabetes induction by PDL1 blockade. We do not observe this in the case of any other congenic strains. This is probably because *Ctla4*and *Il2*both modulate the survival and function of Treg cell population and the blockade of PD1-PDL1 pathway is not enough to limit the ability of these Tregs, and tolerance is maintained in the *Idd3/5* congenic mice. It is also worth mentioning that PDL1 and *Ctla4*mediated tolerance induction functions through two distinct pathways. Allelic interaction of *Ctla4* with the other candidate genes in the *Idd3/5* strain is able to overcome the effect of anti PDL1 treatment and maintain tolerance in these congenic mice. *Slc11a1* plays an important part in antigen presenting function of DCs and may play a role in inducing tolerance to self antigens. *Acadl* is proposed to have a significant role in T cell function and survival by altering fatty acid metabolism. We therefore suggest that the combination of these four candidate genes and their interaction renders the *Idd3/5* congenics resistant to diabetes induction by PDL1 blockade. The *Idd3/5* mice have also been shown to be resistant to other experimental autoimmunity [41].

The *Idd10/18* strain has an insulitis rate of 78%, and ∼50% rate of spontaneous diabetes development [37], in contrast to the *Idd3/10/18* strain which shows greater protection, with 19% of mice developing insulitis and 7% developing type 1 diabetes. The *Idd3/10/18* strain demonstrates a median protection against anti PDL1 accelerated diabetes development with a 31% cumulative incidence as opposed to a cumulative incidence of 94% in *Idd10/18,* and 0% in the *Idd3/5* strain following treatment. The *Idd3/10/18* strain is almost completely protected from diabetes, similar to the *Idd3/5* strain. However, the effect of PDL1 blockade results in significantly different outcomes in respect to diabetes induction. These data also imply that in the absence of negative costimulation by the PD1-PDL1 pathway, CTLA4 possibly maintains the self tolerance with the help of IL-2 (one of the candidate genes in *Idd3*) in case of the *Idd3/5* strain. And IL-2 alone is not sufficient to prevent anti PDL1 mediated accelerated diabetes in case of the *Idd3/10/18* strain. Similarly in *Idd3/5.2* and *Idd3/5.3* strains where the congenic *Idd5.1* locus containing the CTLA4 gene is absent, IL-2 alone cannot prevent diabetes induced by PDL1 blockade.

The *Idd9* strain including sub-congenic *9.1, 9.2 and 9.3* shows high levels of pancreatic infiltrates (90%), but does not develop diabetes at a high rate (3%) [21]. Nonetheless, this strain develops insulin autoantibodies [27]. The *Idd9.1* region was identified to control type 1 diabetes development through TNF-α [42]. *Idd9.2* and *Idd9.3* regions were found to be responsible for preventing the expansion of islet specific CD8^+^ T cells, providing an explanation for the dichotomy of high insulitis incidence and a low rate of actual diabetes development in the *Idd9* strain [24].

Remarkably, a profound increase of diabetes incidence (3% to 50%) was observed after PDL1 blockade in *Idd9* congenic mice. We studied the effect of anti PDL1 on *Idd9* subcongenics (*Idd9.1*, *Idd9.2* and *Idd9.3*). Interestingly, almost 100% of *Idd9.1* and *Idd9.3* mice developed accelerated diabetes following anti PDL1 treatment, in contrast to the *Idd9.2* strain, which was partially protected (56% became diabetic). Findings in the *Idd9* strain suggest that in mice that already have infiltrating lymphocytes in target organs at 10 weeks of age, like *Idd9.1* and *Idd9.3* strains, diabetes development is exacerbated following anti PDL1 treatment. *Idd9.1* and *Idd9.3* strains which develop accelerated diabetes, also show slightly higher scores of insulitis. *Idd9.2* mice had significantly less pancreatic infiltrates and only 56% developed diabetes after anti PDL1 treatment.

The *Idd9* mice strain similar to *Idd3/5* and *Idd3/10/18*, is also resistant to spontaneous diabetes occurrence. However, the level of insulitis is much higher in the *Idd9* strain in comparison to that of *Idd3/5* and *Idd3/10/18*. The difference in the pathogenicity of the disease in the *Idd9* strain is attributed to the Th2 type cellular response induced by the TNFR superfamily gene cluster present in the *Idd9* locus. The combination of three sub loci *Idd9.1, Idd9.2* and *Idd 9.3* renders this strain resistant to the disease even though individually the candidate genes are susceptible. PDL1 blockade negates this interaction and in the absence of any other functional negative costimulatory pathway, disease progression is accelerated and pathogenicity is altered. Among the three subloci of *Idd9, Idd9.2* is considered the most potent region in providing protection against the disease by restraining autoreactive CD8+ T cells. Blockade of PDL1 is known to cause CD8+ T cell exhaustion. This explains the similarity in the anti PDL1 induced diabetes incidence (approximately 50%) in the *Idd9* and *Idd9.2* strains; whereas the *Idd9.1* and *Idd9.3* strains are completely susceptible (90-95%) to anti PDL1 induced diabetes. We also observe a very low frequency of pancreatic infiltrates in the *Idd9.2* strain. This can be explained by the fact that the strains of *Idd9* and its sub regions have a low frequency of autoreactive CD8^+^ T cells in comparison to the NOD mice. Further, the genes in the *Idd9* sub regions prevent a massive expansion of these autoreactive CD8^+^ T cells during disease onset and progression [25]. However, extensive insulitis was observed in the group of *Idd9.2* mice that become diabetic after PDL1 blockade suggesting that in some of the mice the low frequency of autoreactive cells can expand when this regulatory pathway is inhibited. The decreased cytokine and chemokine production in *Idd9* and *Idd9.2* mice may also be related to the low frequency of autoreactive T cells [25] that affects the quality of the infiltrating cells following PDL1 blockade as compared to mice not having protective alleles at *Idd9.2*.

Our study using treatment with anti PDL1 mAb indicates that sufficient numbers of effector cells are present in these congenic strains to mediate type 1 diabetes. The rapid onset of diabetes in some of the *Idd* congenic strains is probably due to auto-aggressive memory/effector T cells that are suddenly set free when PDL1 is blocked, as has been shown in a study in NOD mice [43].

Further, CD4^+^ Type II NKT cells were shown as regulators of diabetes and it was shown that these cells were sufficient in down-regulating diabetes, promoting activity of CD4^+^ BDC2.5 tg T cells *in vivo*. Interestingly, blockade of ICOS and PDL1 was found to negate the regulatory effect of the CD4^+^ Type II NKT cells in the pancreatic lymph node leading to a sudden development of diabetes [44].

We used the *Idd3/10/18* strain to further dissect anti PDL1 mediated diabetes. BDC2.5 Tg CD4^+^ T cells were transferred into NOD and *Idd3/10/18* mice treated with anti PDL1. Similar rates of T cell proliferation were observed in pancreatic LNs of both strains. Corresponding to CD4^+^ TCR Tg T cells tested above, CD8^+^ 8.3 TCR Tg^+^ T cells divided more frequently, as seen by an increase in the number of CFSE-diluted CD8^+^ T cells in *Idd3/10/18* mice (and NOD mice) that received anti PDL1 antibody as compared to mice that received control IgG. These data are similar to our findings in regular NOD mice [4] and suggest that the lower susceptibility of *Idd3/10/18* mice to develop type 1 diabetes following PDL1 blockade is probably not dependent on expansion of CD4 and CD8 T cells, rather that anti PDL1 treatment likely affects behavior of these cells which contributes to lowering susceptibility for developing disease. The role of PDL1 expression in the pancreas and its effect on resistance in this strain remains to be further investigated.

Correlation between the level of basal insulitis and the development of anti PDL1 induced diabetes also proved to be true in the case of *Idd3/5* mice to which anti PDL1 was administered. These mice stayed diabetes free. The *Idd3/5* strain exhibits profound resistance, has the lowest spontaneous diabetes incidence, and also shows the lowest levels of insulitis among all *Idd* congenics.

Prominent genes associated with *Idd3/5* congenic strain are *Il2* and *Il21* from *Idd3*, and *Ctla4* from *Idd5* regions [37]. The role of IL-2 in diabetes has been previously demonstrated. NOD mice express less IL-2 than diabetes resistant mouse strains [45], and low dose IL-2 administered at the onset of type 1 diabetes can reverse established disease in NOD mice [46]. This mechanism has been attributed to an increase in regulatory T cell numbers in the pancreas, and to increased expression of FoxP3, CD25, CTLA-4, ICOS and GITR [46]. Lower levels of IL-2 were found to have an impact on antigen presenting cells like DCs, since low IL-2 levels correlated with higher numbers of DCs and increased T cell stimulation and activation. The cellular mechanism of protection from T1D in *Idd3/5* congenic mice strain is already defined by Hamilton-Williams et al. [9].

Further analysis of the *Idd3/5* region showed that removal of protective alleles at a subcongenic region from the *Idd3/5* region as in the *Idd3/5.2/5.3* strain results in a 15% incidence of disease upon anti PDL1 treatment in contrast to no incidence of disease in the *Idd3/5* group, which supports the role of *Ctla4* at the *Idd5.1* locus in preventing diabetes in a concerted interplay with *Idd3*. Although *Idd3/5.2/5.3* mice do not develop diabetes, an increase of insulitis in *Idd3/5.2/5.3* mice as compared to *Idd3/5* has been reported [33]. These observations support the hypothesis that the ability of PDL1 to accelerate diabetes relies on some minimal amount of effector cell accumulation that is normally manifested as at least a mild insulitis.

## Conclusion

Taken together, our data show that PDL1 blockade destroys the genetic protection mediated by different protective alleles. We show a link between occurrence of insulitis and disease susceptibility through a break of tolerance induced by anti PDL1. We suggest that the presence of a functional CTLA4 allele is probably responsible to prevent disease susceptibility induced by anti PDL1. Increased understanding of the mechanisms of gene-gene interaction, and discovering additional traits that play a role in type 1 diabetes will help identify novel treatments of this disease. The PD1-PDL1 pathway is currently studied for developing therapy for cancer and infectious diseases including HIV, since blockade of this pathway results in increased immune responses against tumor cells [1], [47], [48] and infectious agents [3], [49], [50]. However, we show that blockade of this pathway interrupts critical tolerance mechanisms that operate to prevent autoimmune diabetes. Acceleration of diabetes following PD1-PDL1 pathway blockade to treat disease underscores the need for caution before proceeding to a widespread use of this form of treatment, especially when used in combination with antiCTLA-4 (Ipilimumab) that is currently approved for use in melanoma. A combined blockade of CTLA4 and PD1-PDL1 will in all probability shift the balance from an effective immune response towards autoimmunity. It is important to note that our group had earlier shown that type 1 diabetes resistant NOR mice, which are congenic for the MHC locus to the NOD mice, did not develop diabetes following anti PDL1 treatment [51]. The fact that these congenic mice were protected against type 1 diabetes post-anti PDL1 treatment suggests that PDL1 blockade may still prove suitable in human patients without HLA alleles associated with autoimmune disease such as type 1 diabetes.

Future research should focus on strategies to exploit enhanced immune responses by blocking the PD-PDL1 pathway and at the same time prevent the development of autoimmune disease as a consequence.
